# Genetic Diversity Analysis of the Chinese Daur Ethnic Group in Heilongjiang Province by Complete Mitochondrial Genome Sequencing

**DOI:** 10.3389/fgene.2022.919063

**Published:** 2022-06-21

**Authors:** Mansha Jia, Qiuyan Li, Tingting Zhang, Bonan Dong, Xiao Liang, Songbin Fu, Jingcui Yu

**Affiliations:** ^1^ Scientific Research Centre, The Second Affiliated Hospital of Harbin Medical University, Harbin, China; ^2^ Laboratory of Medical Genetics, Harbin Medical University, Harbin, China; ^3^ Key Laboratory of Preservation of Human Genetic Resources and Disease Control in China, Harbin Medical University, Ministry of Education, Harbin, China; ^4^ Editorial Department of International Journal of Genetics, Harbin Medical University, Harbin, China

**Keywords:** Daur ethnic group, mitochondrial DNA, genetic diversity, maternal inheritance, population genetics

## Abstract

Mitochondrial DNA (mtDNA) has the characteristics of maternal inheritance, high mutation rate, high copy number, and no recombination**.** As the most powerful tool for studying the origin and evolution of modern humans, mtDNA has great significance in the research of population genetics and evolutionary genetics. Here, we provide new insights into the maternal genetic history of the Daur ethnic group by generating complete mitochondrial genomes from a total of 146 Daur individuals in China. We also collected the published complete mitochondrial genome sequences of 5,094 individuals from 56 worldwide populations as reference data to further explore the matrilineal genetic landscape of the Daur ethnic group. First, the haplotype diversity was 0.9943 ± 0.0019 and nucleotide diversity was 0.0428 ± 0.0210. The neutrality tests of the Daur group showed significant negative values and the mismatch distribution curve was obviously distributed in a unimodal pattern. The results showed that the Daur ethnic group has high genetic diversity and may have experienced recent population expansion. In addition, the main haplogroups of the Daur population were haplogroup D (31.51%), M* (20.55%), C (10.28%), F (7.53%), and B (6.85%), all of which were prevalent in northern China. It probably implies the northern Chinese origin of the Daur population. The PCA, *F*_ST_, and phylogenetic analysis results indicated that the Daur group formed a cluster with East Asian populations, and had few genetic differences with the populations in northern China. More importantly, we found that disease-related mutation sites of the mitochondrial genome may be related to ethnic groups, which may have important implications for the prevention and occurrence of specific diseases. Overall, this study revealed the complexity and diversity of the matrilineal genetic background of the Daur ethnic group. Meanwhile, it provided meaningful data for the research on the diversity of the human genome.

## 1 Introduction

Mitochondrial DNA (mtDNA) is the only DNA that exists outside the nucleus of human cells. MtDNA has lower molecular weight and higher mutation rate than nuclear DNA (Chatterjee et al., 2006). Mitochondria have unique cell dynamics to ensure their correct distribution in dividing cells and high fidelity of genomic inheritance through maternal transmission (Mishra and Chan, 2014). Moreover, mtDNA also has a high copy number and lack of recombination properties. MtDNA reveals regional and ethnic genetic differences, and it is widely used in the fields of population genetics, forensic science, and evolutionary anthropology (Zheng et al., 2011; Chaitanya et al., 2016; Font-Porterias et al., 2018). The study of mtDNA genetic markers reflects the evolutionary history of a population, which is helpful to infer the maternal origin of the population and analyze the migration trajectory. Meanwhile, it also reflects the genetic relationship among different populations.

The Daur ethnic group is one of the minority nationalities in northern China, mainly distributed in the Daur Autonomous Banner of Morin Dawa, Inner Mongolia Autonomous Region, and Qiqihar, Heilongjiang Province. The Daur language belongs to the Altaic language family. The Daur nationality is sparsely populated, and there are few studies on its mtDNA polymorphism. The few previous studies available have never performed complete mitochondrial genome sequencing of the Daur population. Therefore, the research on this subject is extremely necessary and significant. In recent years, there have been increasing studies on the genetic polymorphism of East Asian populations, especially Chinese ethnic groups (Park et al., 2017; Trejaut et al., 2019; Wei et al., 2020). However, there are still some ethnic groups that have rarely been studied. It has led to incomplete mtDNA databases for some populations around the world, which has greatly restricted the study of human evolution and origin. The Daur population is a rare ethnic minority in China; therefore, our research samples are extremely precious, and it is necessary to study their genetic diversity. It may be of great significance to study the historical migration and evolution of the East Asian population.

Many researchers pay more attention to the analysis of mitochondrial hypervariable region sequences. However, the mutations in the coding region also make an important contribution to the construction of maternal lineages. Therefore, sequencing of the whole mitochondrial genome will significantly improve the resolution for distinguishing differences between individuals or groups (Seo et al., 2015). On the other hand, the genetic information in the mitochondria can be obtained more accurately and comprehensively. MtDNA has an extremely crucial significance in the related research of population genetics. In this study, we chose the whole mitochondrial sequencing to research the mtDNA diversity of the Daur ethnic group, which would clarify the distribution characteristics of polymorphism sites and haplogroups for the Daur ethnic group and would reveal its maternal genetic structure. The genetic discrepancy between the Daur ethnic group and other populations would also be studied. It would provide powerful genetic information for understanding the history of changes among groups.

## 2 Materials and Methods

### 2.1 Sample Collection

A total of 146 samples were collected from unrelated healthy individuals of the Daur ethnic group in Qiqihar, Heilongjiang Province, including 71 males and 75 females. All of them have lived in Heilongjiang region for at least three generations according to the narrative. Written informed consent was obtained from all participants. This research was approved by the ethics committee of the Second Affiliated Hospital of Harbin Medical University (Approval Number: KY2020-250). All methods were performed in a manner consistent with the approved protocols and in accordance with the relevant guidelines and regulations for human subjects research.

### 2.2 DNA Extraction, Long-PCR Amplification, and Sequencing

DNA was extracted from sample blood using the QIAamp DNA Blood Mini Kit (QIAGEN) according to the manufacturer’s protocol.

At first, six pairs of primers were used for long-PCR amplification to enrich the mitochondrial genome. The primers are described in detail elsewhere (Xu et al., 2021). A total volume of 20 μL in the PCR reaction system containing 2.4 μL 2.5 mm dNTP, 1 μL each of reverse and forward primers (1 μm), 1 μL template DNA (2 ng/μL), 10.2 μL ddH2O, 4 μL 5× TransStart FastPfu Fly Buffer, and 0.4 μL DNA polymerase. PCR was performed under the following cycle conditions: 95°C for 10 min; followed by 28 cycles of 94°C for 20 s, 68°C for 6 min; a final extension at 72°C for 12 min, and hold at 4°C. Amplification products were purified and fragmented. End-repair, end tail, and adapter ligation for fragmented DNA were performed using the NEBNext^®^ DNA Library Prep Reagent Set for Illumina^®^. Library fragment selection and quality assessment were done using Agilent 2100 Bioanalyzer. Finally, the libraries were sequenced in a 2 bp × 150 bp paired-end mode on the Illumina Hiseq platform.

### 2.3 Sequencing Data Processing

The original data was obtained by high-throughput sequencing. By using the MEM algorithm of BWA software (http://bio-bwa.sourceforge.net/) (Li and Durbin, 2010) to compare the original data of each sample with the reference genome, acquired the preliminary mapped results in the BAM file. The human reference genome of this research was the revised Cambridge Reference Sequence (rCRS) of hg38 at UCSC. Picard software (https://broadinstitute.github.io/picard/) was used to analyze the mapped information of each sample, including the ratio of duplicate reads resulting from PCR amplification and the average sequencing depth, etc. GATK (https://software.broadinstitute.org/gatk/best-practices/) (McKenna et al., 2010) was used to calibrate the preliminary mapped results obtained by BWA software, which greatly reduced the false positives and false negatives generated during the sequencing and mapping process. Detected the mutation sites of the complete mitochondrial genome by the GATK Mutect2+HaplotypeCaller method. Information annotation for all variant sites by ANNOVAR (http://annovar.openbioinformatics.org/en/latest/) (Wang et al., 2010). Meanwhile, deep filtering by Perl scripts was performed to obtain detailed mutation information of all samples. Finally, the mitochondrial sequences in FASTA format were generated.

### 2.4 Analysis of Mitochondrial Sequences

To further describe the complex matrilineal genetic landscape of the Daur ethnic group, this study sequenced and generated 146 mitochondrial sequences of the Daur group. In addition, we also searched a total of 5,094 complete mitochondrial sequences from 56 populations as reference data by two researchers. Among them, a total of 2,503 individuals from 26 populations were collected from the 1000 Genome Project. The whole mitochondrial sequences of the 30 populations were screened from published studies and then downloaded from GenBank (https://www.ncbi.nlm.nih.gov/genbank/). Ultimately, there are 5,240 mitochondrial genomes from 57 populations included in our research. Detailed information on the worldwide populations and cited references are listed in Supplementary Table S1.

All of the complete mtDNA sequences in the FASTA format were aligned with rCRS using BioEdit software (Anderson et al., 1981; Andrews et al., 1999). The genetic diversity indexes containing a number of polymorphic sites (S), the total number of mutations (Eta), and the number of haplotypes (h) were calculated by DnaSp v6 software. Nucleotide diversity (Pi), haplotype diversity (HD), the mean number of pairwise differences, neutrality tests including Tajima’s D and Fu’s Fs, and the values of mismatch distribution analysis were estimated using Arlequin ver 3.5.2.2. Analysis of molecular variance (AMOVA) and pairwise fixation index (*F*_ST_) were also generated by Arlequin ver 3.5.2.2. The haplogroups of the complete mitochondrial genome sequences for the Daur group were classified using HaploGrep2 based on PhyloTree build 17 (http://www.phylotree.org/index.htm). In addition, the haplogroups of 56 reference populations worldwide involved in this study have also been redefined. The frequencies of the mitochondrial haplogroups were calculated by direct counting. To reveal the relationship between mitochondrial polymorphism sites and disease of the Daur population, we annotated disease information based on MITOMAP (https://www.mitomap.org/) for variant sites. The chart of sequencing quality, pheatmap, and principal component analysis (PCA) were generated by R 4.0.3. The R packages used for PCA were “tidyr,” “dplyr,” and “ggplot2”. The phylogenetic tree was produced by MEGA. To reconstruct the demographic history for Daur samples, we performed a Bayesian skyline plot (BSP) using BEAST 1.8.4. The plot was visualized with Tracer v1.7.2.

## 3 Results

### 3.1 Sequencing Quality Analysis

**FIGURE 1:**
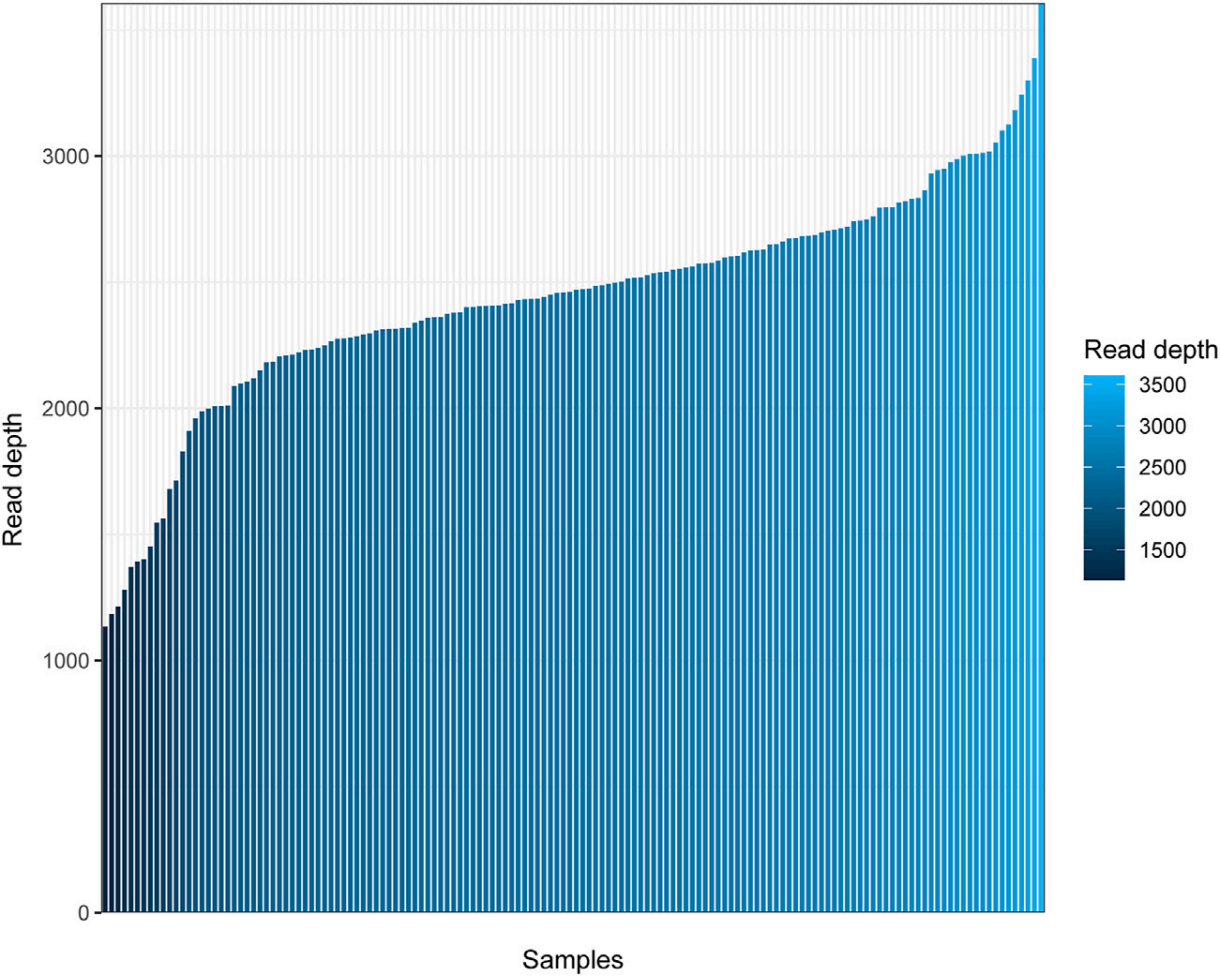
Read depth for the complete mitochondrial genome of 146 Daur individuals. The horizontal axis represents the different individuals sorted from small to large according to the mean sequencing depth, and the vertical axis represents the average read depth.

In the present study, 146 Daur individuals were sequenced successfully. To observe the sequencing quality clearly, we plotted a bar chart to show the depth of sequencing for all individuals. As shown in Figure 1, the sequencing depth of all individuals was higher than 1,100 ×, and approximately ranged from 1,134 × to 3,607 ×. The average read depth was 2,439 × ± 434× (mean ± SD) for each individual. Q20 and Q30 values of 146 sequencing samples are displayed in Supplementary Table S2. The sequencing performance was excellent for the whole mitochondrial genome in our research.

### 3.2 Genetic and Variation of Mitochondrial DNA

To research the genetic and variation characteristics of the Daur ethnic group, we calculated the related genetic diversity indexes (Table 1). A total of 497 variants were observed at 490 positions in the range of the complete mitochondrial genomes, including 77 transversions and 420 transitions. Meanwhile, among 146 Daur individuals analyzed in this study, 111 different haplotypes were detected and 91 of them were unique. It was worth noting that the most frequent haplotype occurred 6 times in all individuals, the following haplotype appeared 5 times and another appeared 4 times. 11 haplotypes occurred 2 times, and six haplotypes occurred 3 times. The haplotype diversity (HD = 0.9943 ± 0.0019) and the nucleotide diversity (Pi = 0.0428 ± 0.0210) were also significant genetic parameters we focused on. The mean number of pairwise differences in the studied Daur population was 20.9604 ± 9.3042, respectively.

**TABLE 1:** Genetic diversity indexes of the Daur ethnic group.

Index	Value
Number of polymorphic sites (S)	490
Total number of mutations (Eta)	497
Nucleotide diversity (Pi)	0.0428 ± 0.0210
Number of haplotypes (h)	111
Haplotype diversity (HD)	0.9943 ± 0.0019
Mean number of pairwise differences	20.9604 ± 9.3042

**FIGURE 2:**
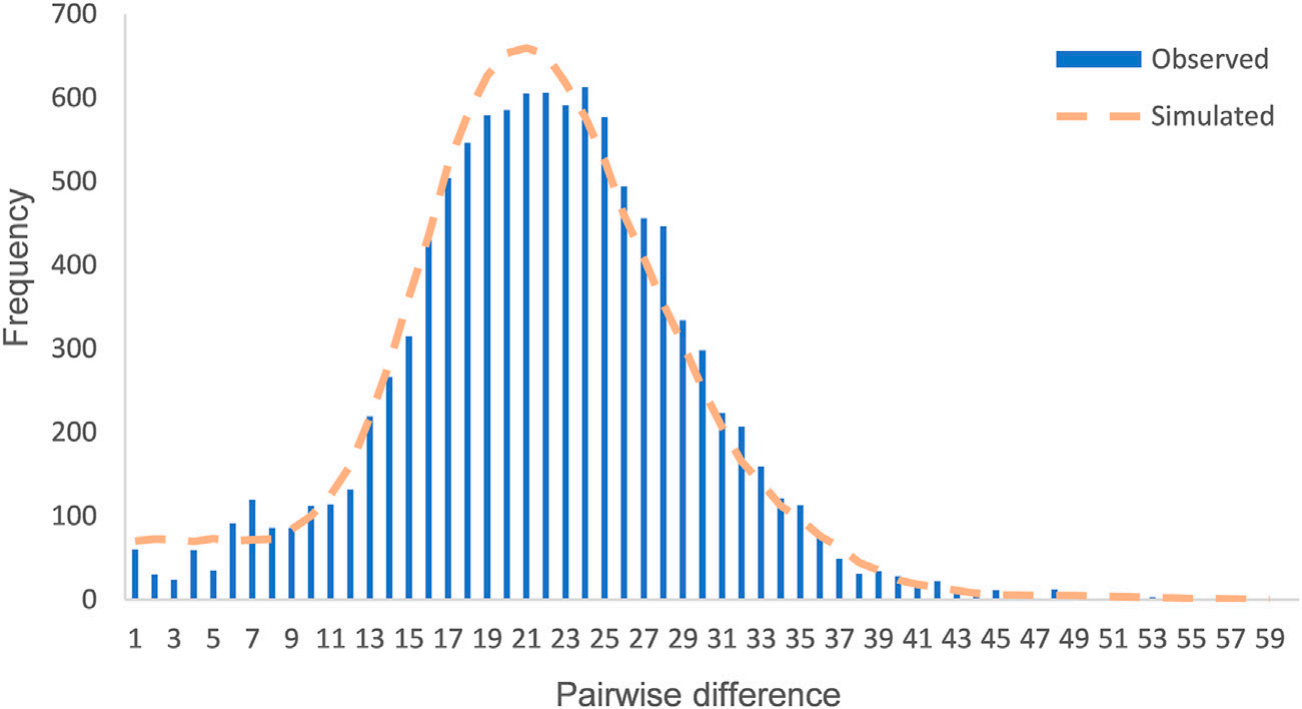
Mismatch distribution of the Daur ethnic group. The orange dotted line represents the simulated model, and the blue bar graph represents the observed model of the Daur ethnic group.

The values of Tajima’s D (−2.503) and Fu’s Fs (−23.820) of the Daur group were calculated. Analysis of mismatch distribution was performed to reflect the historical dynamics of the Daur group, as shown in Figure 2. The mismatch distribution curve of the Daur was obviously distributed in a unimodal pattern. Moreover, we detected the observation model was basically consistent with the expected expansion model. The results implied that the group has experienced expansion or continued growth in the past. The reliability of the result of the mismatch distribution analysis was evaluated through two parameters: sum of squared deviations (SSD) and Harpending’s Raggedness index (HRI). The SSD (0.0003, *p* = 0.940) and HRI (0.0007, *p* = 0.990) of the Daur group showed the statistical test was not significant; it suggested that the hypothesis of group expansion cannot be rejected.

### 3.3 The Population Expansion Time of Daur Ethnic Group

**FIGURE 3:**
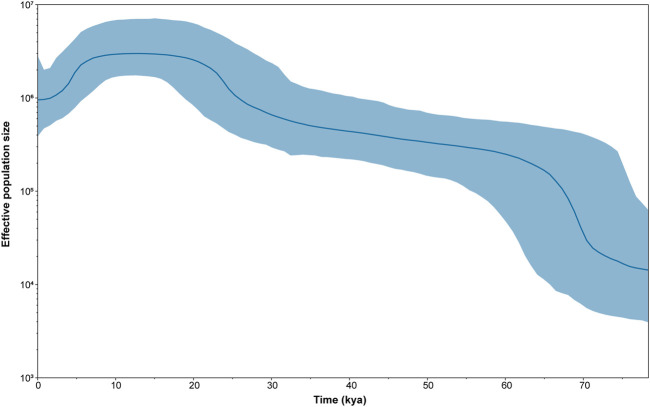
The Bayesian skyline plot (BSP) of changes in effective population size through time for the Daur ethnic group. The dark blue line represents the median population, and the blue line demarcates the boundaries of the 95% highest posterior density.

In order to further verify whether population expansion occurred in the Daur group, we performed the Bayesian skyline plot (BSP) to clarify the population expansion time. Figure 3 shows the effective population size of the Daur group over the past 80 kya. The results showed that the Daur group experienced a significant population expansion around 70 kya, resulting in a sharp increase in population size. The effective population size was relatively stable from 67 kya to 26.6 kya, showing a slow upward trend. After this period of time, the Daur ethnic group experienced a small population expansion at 26.6 kya. After stabilizing for a while, there was a small shrinkage in population size at 7.5 kya.

### 3.4 Mitochondrial Haplogroup Distribution

**FIGURE 4:**
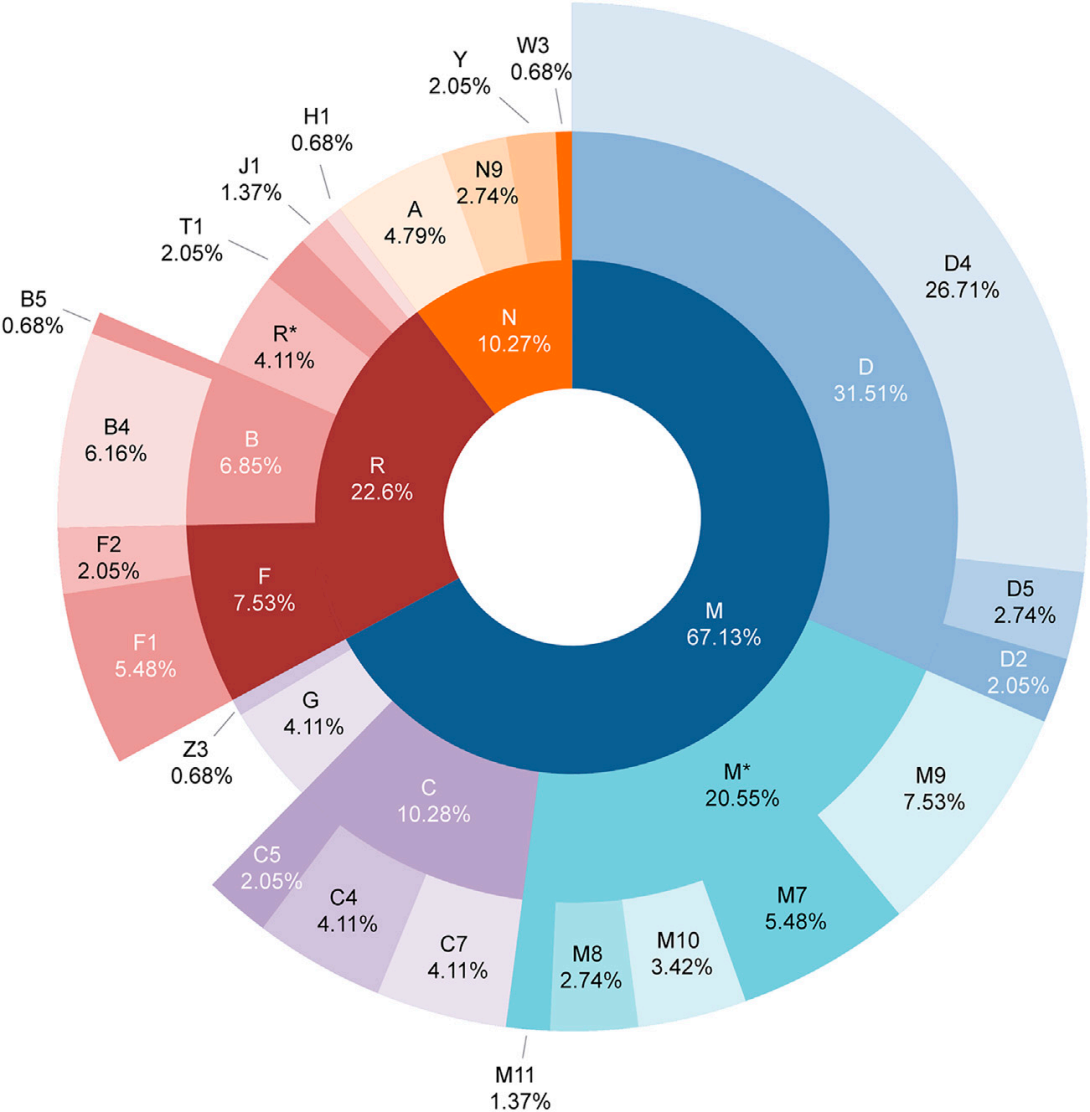
Distribution of mitochondrial haplogroups in the Daur ethnic group. The macro haplogroups M, N, and R displayed in the innermost circle are represented by different colors. The circle in the middle represents the distribution and proportion of each haplogroup belonging to macro haplogroups M, N, and R. The outermost circle shows the distribution and proportion of sub-haplogroups in more detail.

The distribution of each haplogroup reflects the basic composition of the genetic structure of a population. A total of 88 sub-haplogroups were classified among 146 complete mitochondrial genomes of the Daur group based on PhyloTree build 17. The detailed haplogroup classification results of each individual are shown in Supplementary Table S3. Haplogroup D (31.51%) was the most common haplogroup and D4 (26.71%) accounted for the largest proportion among them, then followed by haplogroup M* (20.55%), haplogroup C (10.28%), haplogroup F (7.53%), and haplogroup B (6.85%), which accounted for 76.71% of the haplogroups in the Daur population, while haplogroups A (4.79%), G (4.11%), R* (4.11%), N9 (2.74%), Y (2.05%), and Z3 (0.68%) accounted for a relatively small proportion. Interestingly, European-specific haplogroups such as T1, J1, H1, and W3 also contributed 4.78% to the maternal genetic structure of the Daur population. We have generated a sunburst chart (Figure 4) to show the distribution of Daur haplogroups more intuitively. It can be observed all of the haplogroups belonged to macro haplogroups M, N, and R, macrohaplogroup M occupied the largest proportion among them. Additionally, we reconstructed the haplogroup tree for the tested Daur group, reflecting the evolution of specific branches during defining sub-haplogroups in detail (Supplementary Figure S1).

### 3.5 Specific Disease-Related Mutation Sites in the Daur Mitochondrial Genome

In order to research the distribution of known disease sites in the entire mitochondrial genome of the Daur population, and to grasp the specific maternal genetic markers of the Daur population more accurately, we screened out disease-related sites with a higher mutation frequency in the Daur population. According to disease information annotation based on MITOMAP, we found 71 reported mitochondrial genome loci related to disease in the Daur population, of which 17 loci with a minimum allele frequency higher than 0.05, accounting for 23.94% of the total detected known disease sites. The information of the 17 loci is listed in Table 2. Most of the mutations at these sites occurred in the coding region, and mutation at six sites occurred in the control region. The most common disease-related locus in the Daur ethnic group was A10398G, with a frequency of 0.7466. On the contrary, G11696A and G6962A were the disease-related locus with the lowest mutation frequency, accounting for only 0.0616.

**TABLE 2:** 17 disease-related sites in the mitochondrial genome of the Daur ethnic group.

Gene	Position	Disease	Count	Frequency
ND3	A10398G	PD protective factor/longevity/altered cell pH/metabolic syndrome/breast cancer risk/ADHD	109	0.7466
CYTB	G15043A	MDD-associated	98	0.6712
CR	T310TC	Melanoma	55	0.3767
ND2	C4883T	Glaucoma	46	0.3151
ND2	C5178A	Longevity; extraversion MI/AMS protection; blood iron metabolism	46	0.3151
RNR2	G3010A	Cyclic vomiting syndrome with migraine	45	0.3082
ATP8	C8414T	Longevity	42	0.2877
ND6	C14668T	Depressive disorder associated	41	0.2808
CR	T16189C	Diabetes/cardiomyopathy/cancer risk/mtDNA copy nbr/metabolic syndrome/melanoma	29	0.1986
CR	G16129A	Cyclic vomiting syndrome with migraine	25	0.1712
CR	A16183C	Melanoma	21	0.1438
CR	C150T	Longevity/cervical carcinoma/HPV infection risk	20	0.1370
tRNA-Pro	T16093C	Cyclic vomiting syndrome	17	0.1164
CR	T195C	BD-associated/melanoma	14	0.0959
ND1	T3394C	LHON/diabetes/CPT deficiency/high-altitude adaptation	11	0.0753
ND4	G11696A	LHON/LDYT/DEAF/hypertension helper mut	9	0.0616
COX1	G6962A	Possible helper variant for 15927A	9	0.0616

### 3.6 Genetic Discrepancy for Daur and Other Populations

#### 3.6.1 Analysis of Molecular Variance

To determine the factors that may play a role in the mtDNA diversity, we performed an analysis of molecular variance (AMOVA) by grouping the 57 studied worldwide populations according to the different classifications (including language dialects and geographic regions) as shown in Table 3. We have observed that whether it is classification of language families or geographic regions, within the populations, variation occupied an extremely major proportion. Variations among groups accounted for the minimum contribution. In the groups by geographic distributions of worldwide populations, variation within populations was 87.35%, among populations within groups was 6.93%, and among groups was 5.73%. For the linguistic family groups of worldwide populations, variation within populations was 87.65%, among populations within groups was 9.65%, and among groups was 2.70%. Moreover, the values of geographic regions were lower than those of the language dialects groups for the variation within populations and among populations within groups. Populations separated by geographic regions contained a higher percentage of variation compared to the groups of language families after grouping.

**TABLE 3:** The AMOVA results based on 57 worldwide populations.

Grouping	Number of populations	Number of groups	Among groups	Among populations within groups	Within populations
Geographic distributions of worldwide populations	57	7	5.73	6.93	87.35
Linguistic families of worldwide populations	57	11	2.70	9.65	87.65

#### 3.6.2 Principal Component Analysis Based on Haplogroup Frequency

**FIGURE 5:**
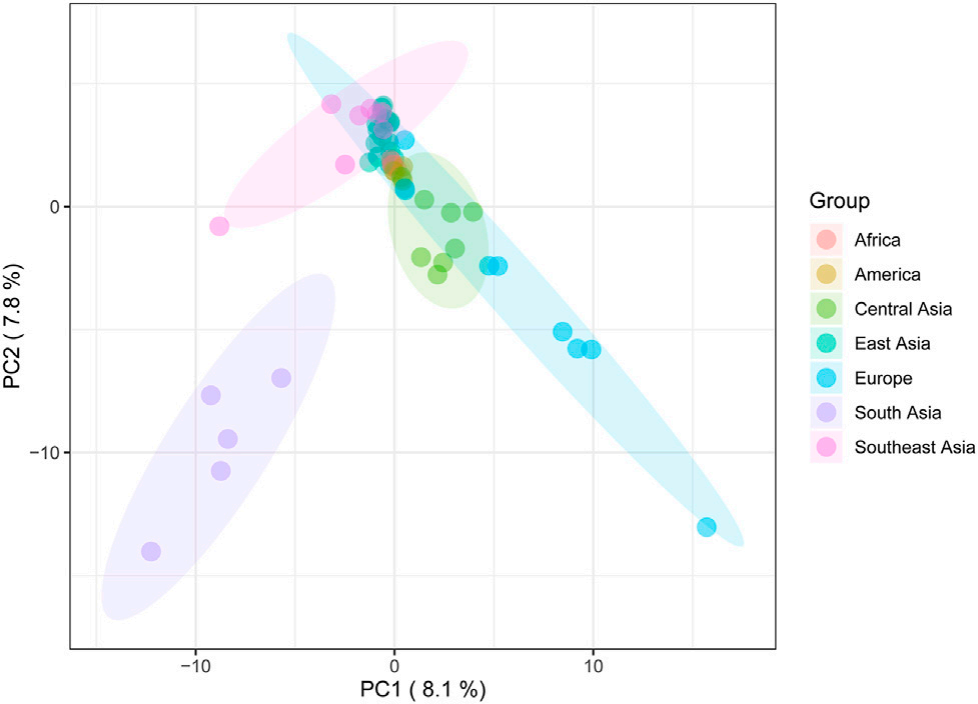
The principal component analysis (PCA) plot for the Daur group and 56 worldwide populations. Daur belongs to the East Asian cluster, indicated in dark green.

To investigate the genetic discrepancy between the Daur ethnic group and 56 other worldwide populations. Principal component analysis (PCA) based on haplogroup frequency was performed (Figure 5, Supplementary Figure S2, Supplementary Figure S3). Due to the higher variation for geographical grouping based on the AMOVA results, we divided the tested populations into seven groups according to the geographical regions. The first three principal components explained 23.7% of the variation, of which PC1, PC2, and PC3 accounted for 8.1%, 7.8%, and 7.8%, respectively. The PCA results showed East Asian populations clustered very tightly in the context of analysis of worldwide populations.

**FIGURE 6:**
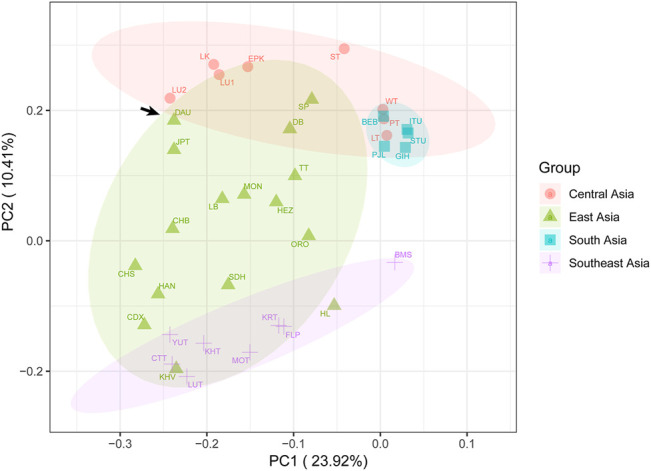
The principal component analysis (PCA) plot for the Daur group and other Asian populations. The Daur group (DAU) is indicated with arrows.

To observe the genetic relationship more clearly between the Daur group and other East Asian populations, we further performed PCA in the context of the entire Asian mtDNA (Figure 6, Supplementary Figure S4, Supplementary Figure S5). A total of 43.73% of genetic variations were extracted by the first three components (PC1: 23.92%, PC2: 10.41%, and PC3: 9.40%). In the PC1 and PC2, the point representing the Daur population was relatively closer to JPT and LU2 (Lowland Uyghur). In the PC1 and PC3, the Daur population was also relatively closer to JPT and LU2. The plot of PC2 and PC3 illustrated the Daur population was closer to some populations living in the Tibetan Autonomous Region, such as DB (Deng), TT (Tingri Tibetan), and SP (Sherpa).

#### 3.6.3 Pairwise Fixation Index (*F*_ST_) Values Reveal Population Genetic Distance

**FIGURE 7:**
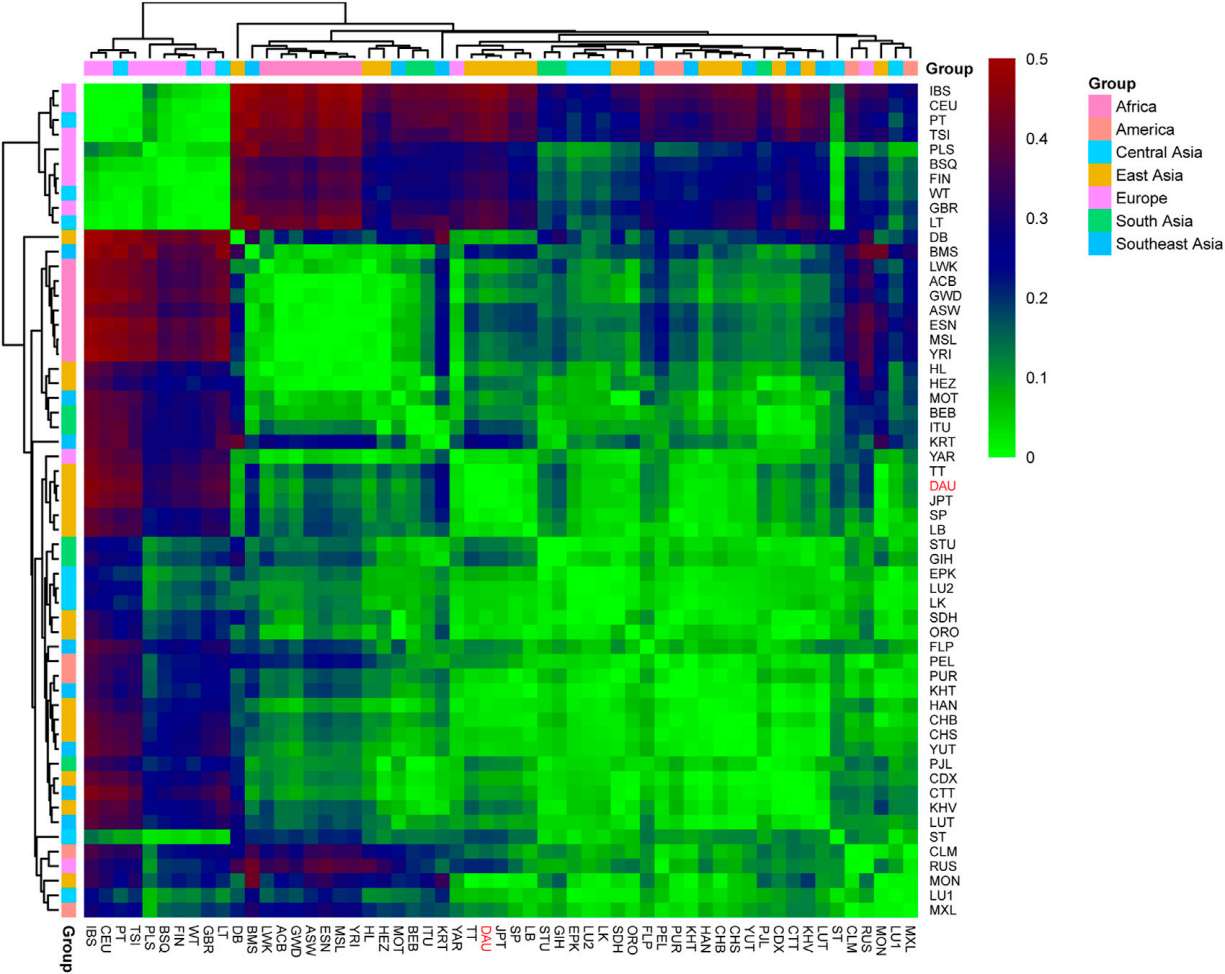
Heatmap for genetic distance among the Daur ethnic group (DAU) and 56 worldwide populations. Visualizing the *F*_ST_ values with different colors. The color red represents the high *F*_ST_ values, and the green represents the low *F*_ST_ values. DAU is highlighted in red.

To reveal the discrepancy in the matrilineal genetic landscape between the Daur ethnic group and other populations, the pairwise *F*_ST_ values for the complete mitochondrial sequences between the Daur group and 56 reference populations were calculated as shown in Supplementary Table S4. Our results showed that the *F*_ST_ values between the Daur group and the reference population ranged from 0.00402 to 0.46263. The lowest pairwise *F*_ST_ value was between the Daur group and TT (*F*_ST_ = 0.00402), followed by JPT (*F*_ST_ = 0.00464). The Daur group also showed lower *F*_ST_ values with SP (*F*_ST_ = 0.01728) and MON (Mongola, *F*_ST_ = 0.01914). In the comparison with the Daur group, the largest value was compared with IBS (Iberian Population in Spain, *F*_ST_ = 0.46263), followed by CEU (Utah Residents with Northern and Western European Ancestry, *F*_ST_ = 0.43967). Meanwhile, all the pairwise *F*_ST_ values were visualized by a heatmap to show the genetic distance more clearly, as shown in Figure 7. Populations were grouped according to the geographic distribution.

#### 3.6.4 Phylogenetic Analysis

**FIGURE 8:**
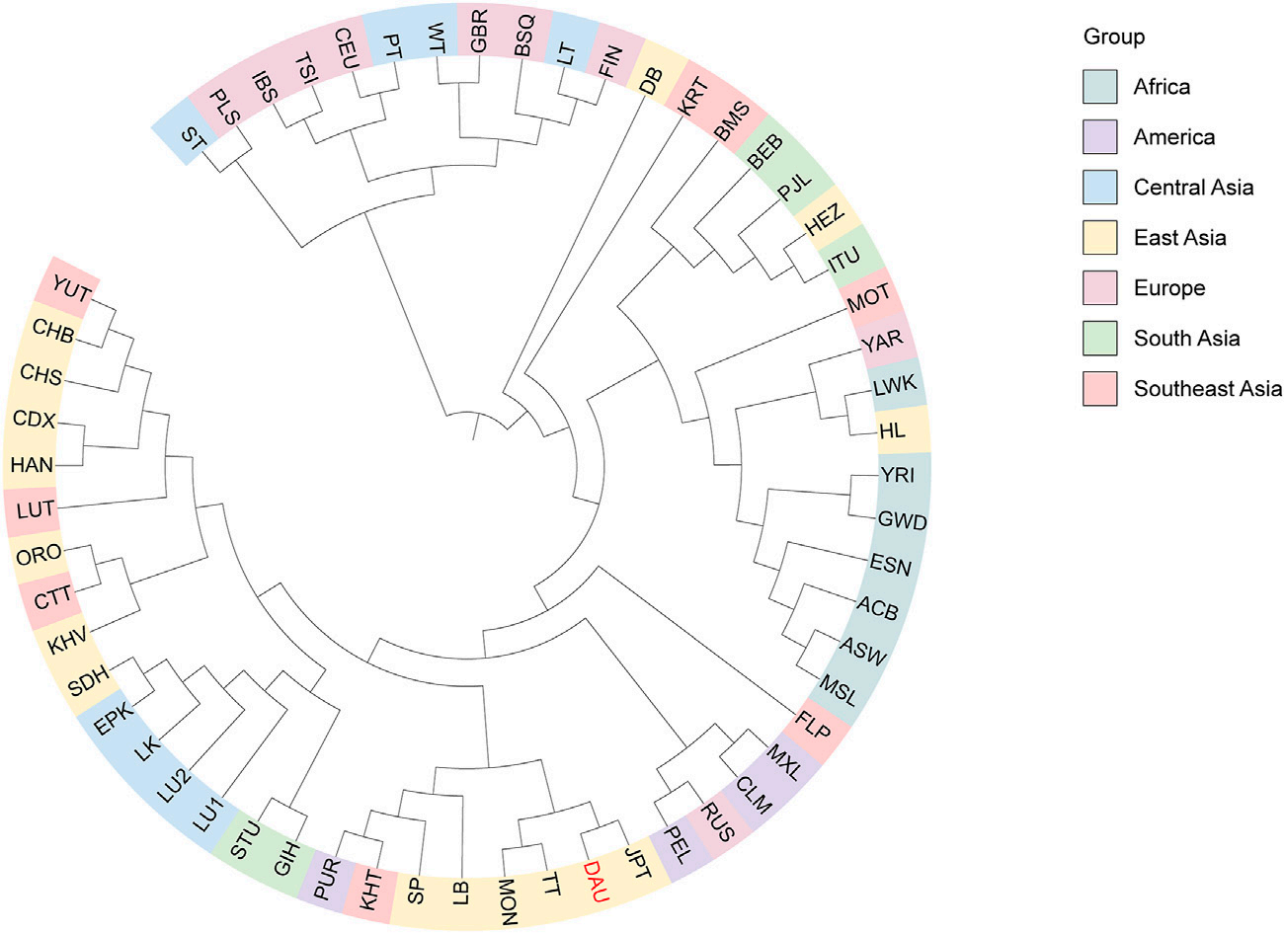
The phylogenetic UPGMA tree for the Duar ethnic group (DAU) and 56 reference populations. The tree is colored according to geographic origins. DAU is highlighted in red.

We conducted a phylogenetic analysis to further clarify the genetic relationship between the Daur ethnic group and other populations and generated a phylogenetic tree based on the *F*_ST_ values (Figure 8). We still divided the 57 populations into seven groups according to their geographical distribution: Africa cluster, America cluster, Central Asia cluster, East Asia cluster, Europe cluster, South Asia cluster, and Southeast Asia cluster. We observed that the Daur population and JPT, MON, and TT gathered on the same subbranch, especially clustering closer to JPT.

## 4 Discussion

Mitochondria, as a useful genetic marker, reflect the characteristics of maternal inheritance and are suitable for population evolution analysis. The main purpose of the present study was to understand the maternal genetic diversity of the Daur ethnic group and to provide important genetic information for the study of human genome diversity.

We analyzed the genetic variation in the complete mitochondrial genomes of the Daur ethnic group from 146 Daur individuals. The haplotype diversity (Nei and Tajima, 1981) is a measure of the uniqueness of a particular haplotype in a certain population, rendering a high gene diversity value (HD = 0.9943 ± 0.0019) in the Daur group. Meanwhile, the value of nucleotide diversity (Nei and Li, 1979) (Pi = 0.0428 ± 0.0210) also revealed that the Daur ethnic group had high genetic diversity and rich genetic resources. The neutrality tests can be used to detect natural selection among the nucleotide sequence variants in a population. In this study, the significant negative values of the neutrality tests (Tajima’s D and Fu’s Fs) mainly reflected that the Daur population experienced the population expansion after a bottleneck recently or indicated an excess of rare variation (Tajima, 1989; Fu and Li, 1993; Carlson et al., 2005). At the same time, the results of the neutrality tests deviated from the neutral mutation significantly. According to the results of the mismatch distribution analysis, on the other hand, it was speculated that the Daur group underwent recent population expansion potentially (Mousset et al., 2004). BSP also proved that Daur had experienced a significant population expansion at 70 kya and a small population expansion at 26.6 kya. Therefore, all of the results reached a consistent conclusion, which further supported the speculation of population expansion powerfully.

Mitochondrial dysfunction and defects caused by mitochondrial DNA polymorphism are related to many diseases. It has been reported that the A10398G variant was probably related to metabolic syndrome (Yan et al., 2014), attention deficit and hyperactivity disorder (ADHD) (Hwang et al., 2017), breast cancer susceptibility (Tengku Baharudin et al., 2012), and Parkinson’s disease (PD) (Jiang et al., 2004). Meanwhile, A10398G may lead to the reduction of the function of the complex I, and the level of reactive oxygen species (ROS) in the cell increased subsequently (Mohamed Yusoff et al., 2018). It will further accumulate more damage to mtDNA to promote the occurrence and development of the disease. It is reported that the generation of ROS may be related to type 2 diabetes mellitus (T2DM) risk (Chalkia et al., 2018). Furthermore, studies have shown that the G11696A mutation may be related to Leber’s hereditary optic neuropathy (LHON) (De Vries et al., 1996; Dai et al., 2018). The mutation frequency of Daur in G11696A was only 0.0616, it is speculated that the Daur population was less susceptible to LHON caused by the mutation of the locus.

The results showed that the Daur ethnic group in this study produced 88 specific sub-haplogroups. The frequency of haplogroups varies with varying degrees among populations in different regions. Previous studies have shown that haplogroup D maintained a very high overall frequency among East Asian, North Asian, and Central Asian populations (Derenko et al., 2007; Derenko et al., 2010). Haplogroup D4 clades more likely reside in the north of East Asia (Zheng et al., 2011). It is also prevalent in northern and northeastern China, implying a potential northern China origin of this haplogroup (Li et al., 2019). Haplogroup M was initially thought to be an ancient marker of East Asian origin. The geographic distribution of M9 is in Central and East Asia, with the highest frequency in Tibet (Chandrasekar et al., 2009). There are significant differences in the frequency of haplogroup M9a between high-altitude Tibetan populations and low-altitude populations, and it has the highest frequency in the Tibetan population (Li et al., 2016). Haplogroup C7 mainly existed in East Asia (Derenko et al., 2010). Haplogroup B4 was a typical haplogroup in southern China (Li et al., 2019). It was worth noting that the most common European mitochondrial haplogroups T, J, H, and W have also been detected in the Daur ethnic group (Grignani et al., 2009; Kozin et al., 2020). These results suggested that most of the haplogroups of the Daur ethnic group were popular in East Asia, and our results proved that the Daur population belongs to East Asian lineage and originated from northern China. In addition, due to the emergence of European-specific haplogroups in the results, we speculated that European ancestry also contributed a small proportion to the maternal inheritance pool for the Daur ethnic group.

AMOVA was used to detect significant variation in the genetic structure of mtDNA among populations (Bodner et al., 2011). We found that when the dominant variation occurred within populations, it revealed more genetic discrepancy within populations. The variation among groups based on geographic regions was slightly higher than that based on linguistic families. The results indicated that geographical grouping might provide a better explanation for the genetic divergence of complete mitochondrial genomes among groups than linguistic grouping.

As for the PCA results, the close clustering between the Daur group and East Asian populations meant that there were almost no genetic differences between them. It confirmed the previous conclusion that the Daur ethnic group belongs to the East Asian branch. In the PCA for all Asian populations, we found the Chinese Heilongjiang Daur group had a close genetic relationship with JPT, TT, SP, DB, and LU2. The population genetic differences between East Asia and Southeast Asia seem to be less obvious to be detected. On the contrary, Daur and South Asian groups performed the farthest genetic relationship among all Asian populations. Meanwhile, the genetic structure of the Daur ethnic group was also well expressed by PCAs.

*F*_ST_ provides important insights into the evolutionary processes that influence the structure of genetic variation within and among populations, and it is among the most widely used descriptive statistics in population and evolutionary genetics. The small *F*_ST_ value means that the allele frequencies in each population are similar. If the value is larger, that means the allele frequencies are different, indicating that the genetic distance is farther (Holsinger and Weir, 2009). According to our results, the Daur population showed a close genetic distance with TT, JPT, SP, and MON. Among them, Daur had the closest genetic distance with TT in the Tibetan region. In addition, the Daur ethnic group with IBS showed the farthest genetic distance among all studied populations. Overall, the Daur ethnic group showed a closer genetic relationship with the vast majority of East Asians, especially the north Chinese populations. However, the Daur group showed obvious genetic divergence with European populations.

At the same time, the phylogenetic tree generated based on the *F*_ST_ values also revealed consistent results. The Daur group apparently congregated with East Asian populations and distributed in the nearest sub-branch with JPT. It revealed that there was little genetic difference between the Daur group and JPT; they had a close maternal genetic relationship. Our results may be due to the Daur sampling site in this study being located in northeast China, which is geographically close to Japan, and the recent introduction of the Daur genes into Japanese or their common maternal origin. In addition, the results revealed that the genetic distance between the Daur group and TT was relatively close. It may be due to the gene exchange and fusion between the Daur population and TT during historical development. The Daur ethnic group and MON also showed a close genetic relationship; they both belong to the Altaic language family. It is reported that the Daur ethnic group originated from the Mongolian ethnic group. The results of this study may further explain the view from the perspective of maternal genetics.

Our research provided a complex and comprehensive maternal genetic landscape of the Daur ethnic group. First, we found that the Daur ethnic group has a high genetic diversity and may have experienced recent population expansion. According to the results, most of the haplogroups of Daur are prevalent in East Asia. It is confirmed that the Daur group belongs to the East Asian lineage and originated from north China. All results of PCA, *F*_ST_, and phylogenetic tree revealed that the Daur group was closely clustered with East Asian populations, especially in northern China. The Daur ethnic group showed a closer genetic relationship with TT, MON, JPT, and SP. We found that the specific disease-related mutation sites of the mitochondrial genome may be ethnic-related. Overall, the mitochondrial genome generated in this study would enrich the existing mtDNA database, actively promoting the research on the genetic diversity and population historical dynamics of the Daur ethnic group.

## Data Availability

The datasets presented in this study can be found in online repositories. The names of the repository/repositories and accession number(s) can be found below: https://www.ncbi.nlm.nih.gov; BankIt2567580: ON127701—ON127846.
