# Supplementary figures and images for: Genetic Diversity Analysis of the Chinese Daur Ethnic Group in Heilongjiang Province by Complete Mitochondrial Genome Sequencing
Source: Front Genet. 2022 Jun 21;13:919063. doi: 10.3389/fgene.2022.919063 (PMC9253502; doi:10.3389/fgene.2022.919063)

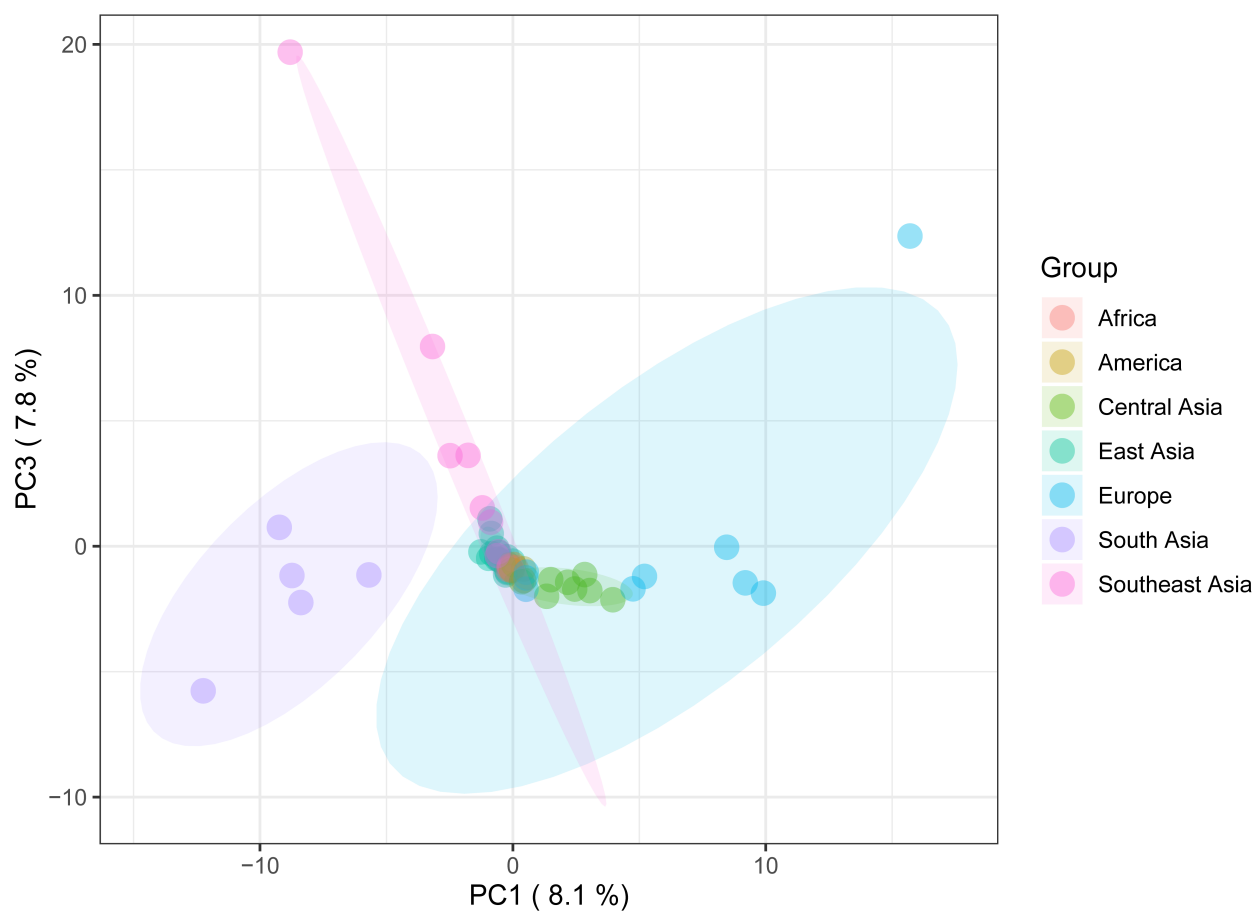

Supplement: Supplementary file 1 [file DataSheet1.ZIP › Supplementary Figure S2.pdf]

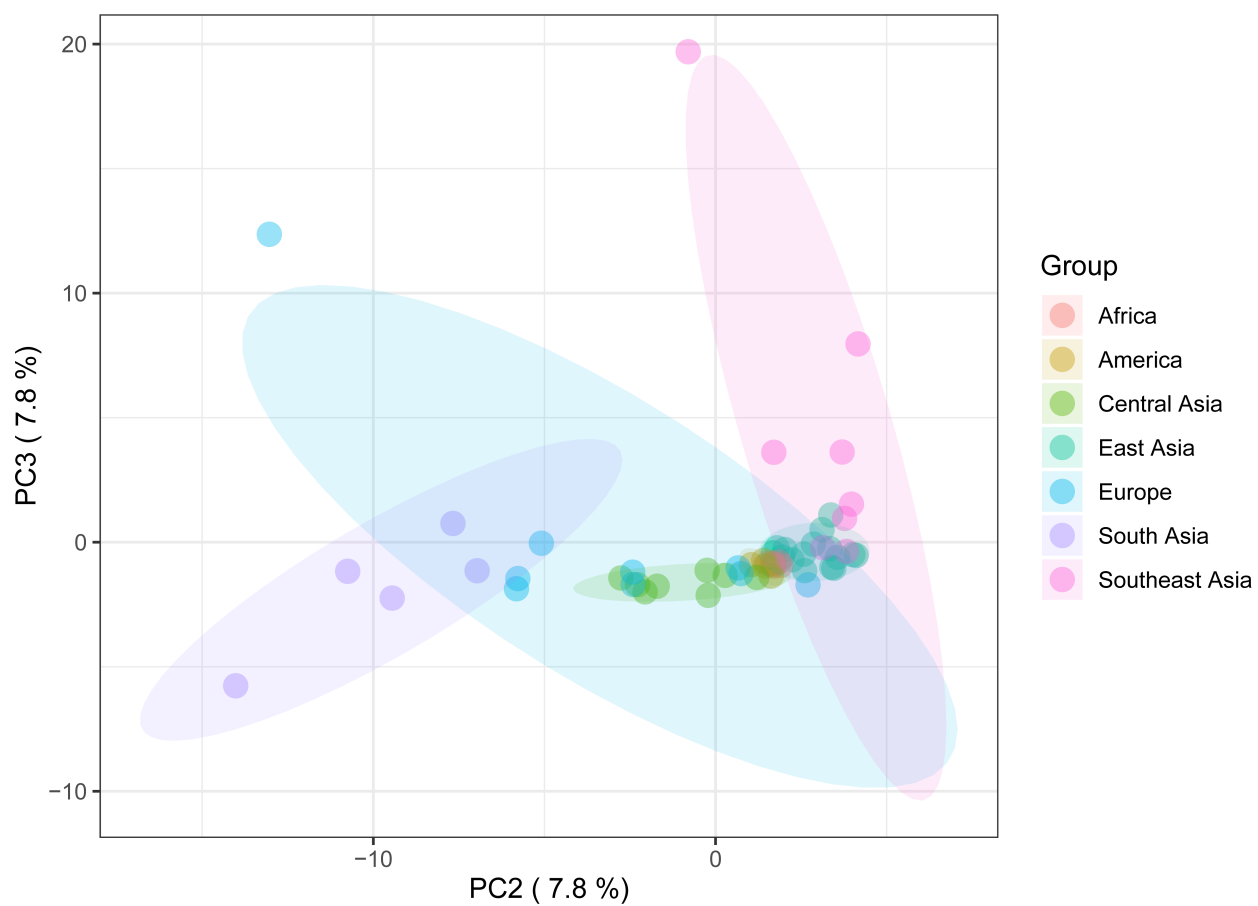

Supplement: Supplementary file 1 [file DataSheet1.ZIP › Supplementary Figure S3.pdf]

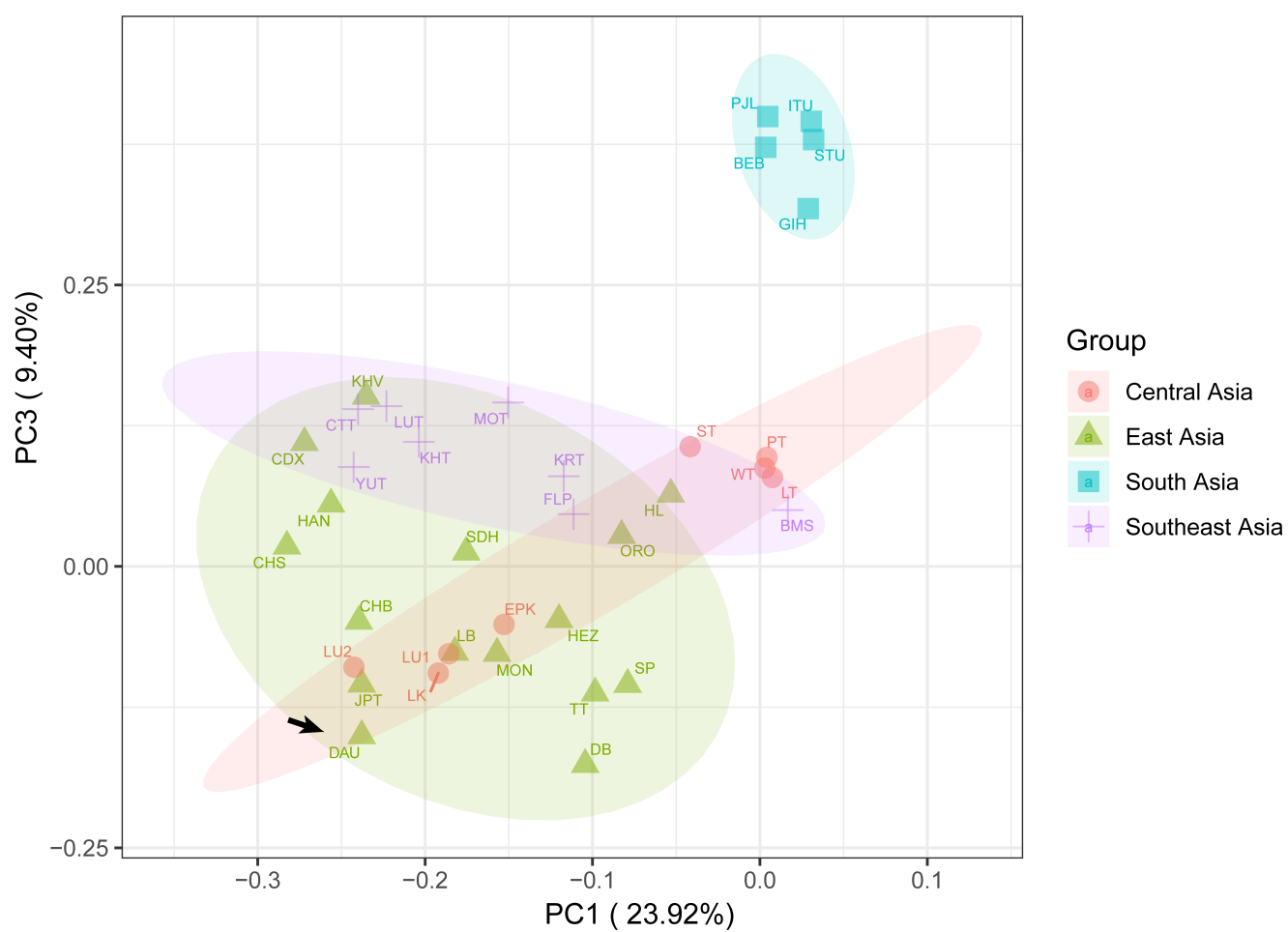

Supplement: Supplementary file 1 [file DataSheet1.ZIP › Supplementary Figure S4.pdf]

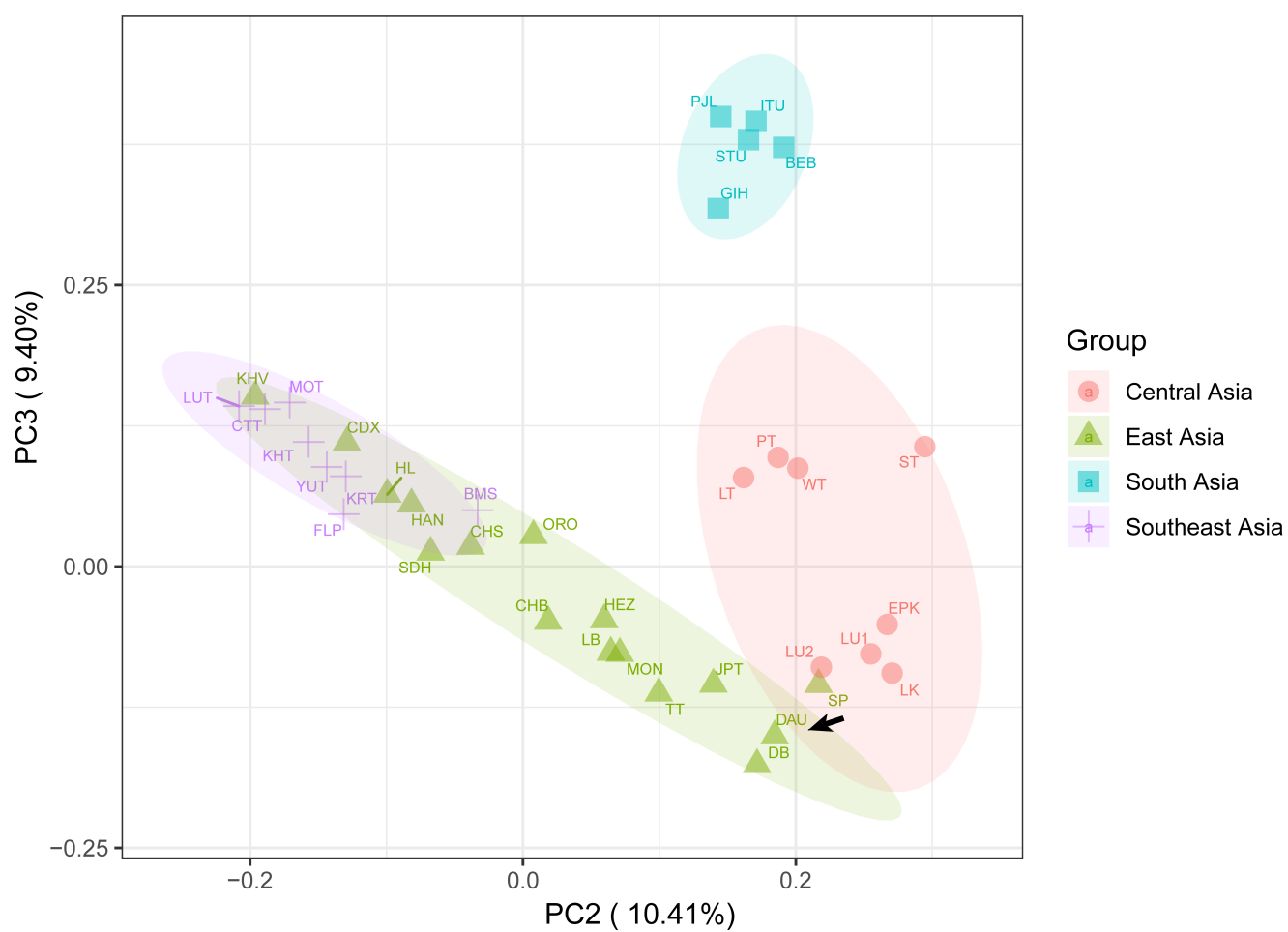

Supplement: Supplementary file 1 [file DataSheet1.ZIP › Supplementary Figure S5.pdf]
