# Supplementary material for: Genetic Diversity Analysis of the Chinese Daur Ethnic Group in Heilongjiang Province by Complete Mitochondrial Genome Sequencing
Source: Front Genet. 2022 Jun 21;13:919063. doi: 10.3389/fgene.2022.919063 (PMC9253502; doi:10.3389/fgene.2022.919063)
Supplement: Supplementary file 1 [file Supplementary Figure S1.pdf › Supplementary Figure S1.pdf]

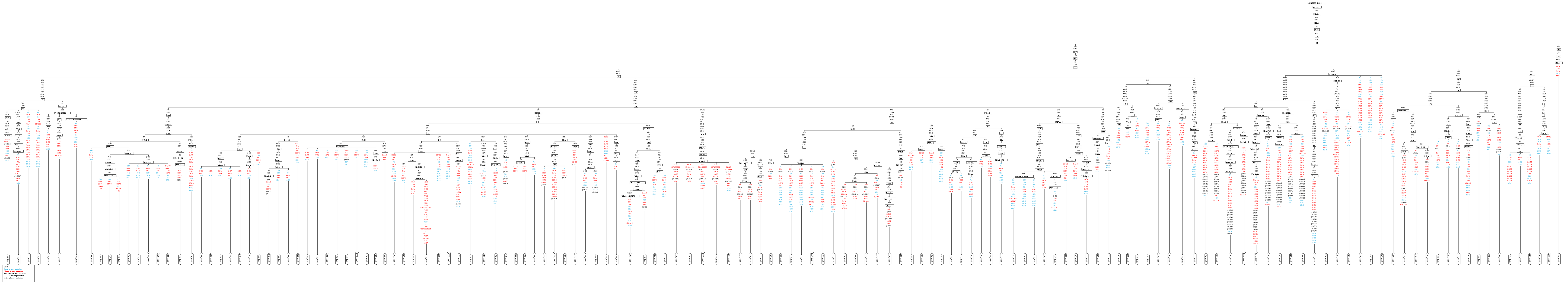
rCRS NC_012920
H2a2a1
263
H2a2a
8860
15326
96_draft
67_draft
172_draft
125_draft
228_draft
117_draft
KEY
Local private mutation
Global private mutation
@ = assumed back mutation
or missing mutation
Heteroplasmic mutation
